# How is automation related to work characteristics and employee well-being? A work design perspective

**DOI:** 10.1177/10519815261488811

**Published:** 2026-10-13

**Authors:** Susanne Tafvelin, Maria Forsgren, Andreas Stenling

**Affiliations:** 1 Department of Psychology, 8075 Umeå University, Umeå, 901 87, Sweden; 2 Department of Leadership and Control & Command, 42748 Swedish Defence University, Stockholm, Sweden; 3 Department of Sport Science and Physical Education, 4343 University of Agder, Kristiansand, Norway

**Keywords:** automation, decision support techniques, artificial intelligence, work, quality of life, social workers

## Abstract

**Background:**

Automation, including both robots and AI, are increasingly implemented in the public sector to handle an increased demand for services while facing a shortage of staff. However, little is known of how automation is related to employees’ work characteristics and well-being.

**Objective:**

The purpose of the present study was to examine how automation is related to work characteristics and well-being in a national sample of social workers.

**Methods:**

A cross-sectional survey was conducted with a sample of 1428 social workers in Sweden, comparing those working with automation technologies (e.g., robotization in terms of automated decision-making support in financial aid) to those without.

**Results:**

The findings indicate that automation is negatively related to relational aspects of work, such as beneficiary contact and social support, and increases perceived workload and role conflict. However, no significant differences were found on decision-making autonomy, role clarity, or skill variety. Despite these work design changes, no significant differences in well-being outcomes, such as burnout and vigor, were observed between the two groups.

**Conclusion:**

The findings suggest that automation may alter the nature of social work without necessarily changing employee well-being. These insights have implications for organizations seeking to implement automation in a way that supports employees’ work experience and well-being.

## Introduction

The integration of automation, including both robots and AI, into working life has the potential to significantly reshape professions, influencing both the nature of work and the well-being of workers.^1^ This is particularly true in the public sector, such as the social services, where robots and AI are increasingly implemented to handle an increased demand for services while facing a shortage of staff.^2^ Studies of the impact of robots and AI on highly educated employees in the public sector such as social workers are still in its infancy, but work design theories provide a valuable framework for examining how automaton affects employees’ work characteristics and for assessing its impact on employee well-being.^3^ The present study therefore sets out to examine how automation in terms of automated decision-making support is related to social workers’ work characteristics and well-being.

### How does automation affect work characteristics?

Work design, a well-established concept in applied psychology and management studies,^4^ encompasses the structure and organization of employees’ tasks, roles, responsibilities, and interactions. It involves determining aspects such as decision-making authority, job composition within teams, and the distribution of diverse tasks within individual roles.^5^ The SMART model of work design, developed by Parker & Knight,^6^ emphasizes five key principles for designing high-quality jobs: stimulating, mastery, autonomous, relational, and tolerable work characteristics. Stimulating work characteristics fosters motivation and engagement through variety and challenge. Mastery work characteristics ensures that work allows skill development and a sense of competence. Autonomous work characteristics refers to autonomy and decision-making control. Relational work characteristics highlight the importance of social connections and meaningful relationships at work. Finally, tolerable work characteristics focus on managing workload and job demands to prevent stress and burnout. The SMART model provides a comprehensive framework for creating enriching and sustainable jobs.

According to Parker and Grote,^3^ automation may have both beneficial and detrimental effects on employees, depending on its effects on these key work design principles. Automation occurs when a machine or technology performs work tasks. There are two types of automation; using robots for automation of manual work tasks, or using AI for automation of work tasks that demands cognitive abilities.^7^Automation may for example affect to what extent social workers perceive their job as *stimulating* as it may affect skill variety, which refers to the range of skills and abilities utilized in one's job.^8^ Automation can enhance skill variety by taking over monotonous tasks,^9^ allowing social workers to engage in diverse and complex activities that utilize a broader skill set. However, there is a risk that automation could deskill the profession if not implemented thoughtfully, leading to roles that are more narrowly defined and less challenging,^3^ a worry expressed by social workers in previous qualitative studies.^10^

Automation may also affect social workers perception of *mastery* in terms of role clarity, which involves understanding one's job responsibilities. On the positive side, automation can streamline processes, standardize tasks, provide clearer guidelines, reducing ambiguity and enhance understanding of job responsibilities.^3^ However, automation can also introduce new complexities, such as the need to adapt to evolving technologies or shifting job roles, which may lead to uncertainty and confusion.^11^ If not properly implemented, automation may blur role boundaries, create conflicting expectations, and increase cognitive demands.^3,12^

Furthermore, automation may also affect social workers’ experience of *autonomy* in terms of for example decision-making autonomy, which refers to the degree of control and discretion employees have over their work tasks and processes.^8^ For instance, the implementation of automated systems may streamline routine tasks, potentially increasing autonomy by allowing social workers to focus on more complex decision-making aspects of their roles.^3^ For example, a recent study showed that air traffic controllers experienced increased autonomy when automation was introduced^13^ However, if automation leads to rigid protocols or reduces the scope of professional judgment, it may diminish autonomy and feelings of control, which has been raised as a concern in previous studies.^10^

*Relational aspects* of the job may also be affected by automation, such as beneficiary contact and support. Beneficiary contact involves direct interactions between social workers and clients,^13^ which are central to the profession. Automation can impact this contact by handling administrative tasks, potentially freeing up time for more meaningful client interactions.^12^ Conversely, over-reliance on automated systems might reduce face-to-face engagements, undermining the relational aspect of social work.^14^ In addition, automation may affect social support in the workplace which includes the assistance and encouragement received from colleagues and supervisors. Automation can influence social support by changing communication patterns and team dynamics. While it may facilitate information sharing through digital platforms,^15^ it could also reduce opportunities for in-person interactions, potentially impacting the social fabric of the workplace.^16^

Finally, automation may affect perceived *tolerable* work characteristics in terms of work demands and role conflict. Workload pertains to the amount of work assigned to an individual within a specific time frame. Some studies suggest that automation may reduce social workers workload by handling administrative repetitive tasks,^10^ potentially alleviating stress and preventing burnout.^3^ However, if automation leads to increased expectations for productivity or adds complexity to the remaining tasks, it may inadvertently increase workload.^17^ Automaton may also introduce role conflict, when there are incompatible demands, if automated systems impose requirements that clash with professional judgment or ethical standards.^3^

### Implications for employee well-being

The interplay between automation and the work characteristics previously described significantly affects employee well-being. In the current study we focus on employee psychological well-being, which is defined as a subjective experience and functioning.^18^ The literature on psychological well-being differentiates between hedonic and eudaimonic well-being. Hedonic well-being includes high positive affect and happiness but also negative aspects such as burnout and depression.^19^ Eudaimonic well-being includes experiences of meaning, fulfillment and thriving.^20^ To include both positive and negative aspects of psychological well-being,^21^ as well as capturing both hedonic and eudaimonic well-being, we study how work characteristics affect burnout (hedonic well-being) and vigour (eudaimonic well-being).^3^ For example, reduced autonomy and increased role conflict can lead to burnout, characterized by emotional exhaustion and diminished personal accomplishment. In line with this, a recent study found that employees working with robots tended to exhibit lower levels of well-being,^22^ and a recent vignette study found that automation was related to increased stress.^23^ Conversely, enhanced skill variety and social support can foster vigor, reflecting high levels of energy and mental resilience. However, a recent systematic review concludes that the impact of automation on employee well-being is fragmented^24^ and studies of automation in relation to eudaimonic well-being is lacking. Also, Demerouti et al.^25^ discuss how job demands and resources influence burnout and engagement, underscoring the need for a balanced work design.

### The present study

Adhering to recent calls in the literature about the need for knowledge on the implications of automation for employees^3,25^ the purpose of the present study was to examine how automation in terms of automated decision-making support is related to work characteristics and well-being among social workers. We contribute to the literature on automation at work by drawing on work design theories to get a fuller understanding of how automation may shape highly educated professions such as social workers, influencing the nature of work and the well-being of workers.^1^ We also add to the literature how automation is related to different types of psychological well-being, examining the association of not only hedonic well-being but also eudaimonic well-being in terms of vigour.

Using data from a large-scale survey study among social workers in Sweden, we compared the perception of work characteristics as well as well-being among of social workers working with and without robotization in terms of automated decision-making support in financial aid. Based on our literature review, we chose to examine work characteristics that represented all five key principles in the SMART model, and we chose one or two factors from each key principle that have been highlighted in previous literature as relevant to automation. In total, we examined seven work characteristics, and these included stimulating aspects in terms of skill variety and use, mastery in terms of role clarity, autonomy in terms of decision-making autonomy, relational aspects in terms of beneficiary contact and social support and finally tolerable demands in terms of work demands and role conflict. We did not specify any a priori hypotheses about the implications of automation on work characteristics and employee wellbeing due to the lack of previous research on the topic. Instead, we consider this an exploratory study with the aim to generate hypotheses for future studies.

## Method

### Procedure and participants

We have conducted a national survey study together with Statistics Sweden among social workers employed in the municipal sector. Participants for this cross-sectional study was sourced from the Swedish occupational register targeting social workers (according to SSYK code 2661) with the reference period being December 2023. The entire population of social workers (N = 17,793) were invited to the survey, and 6882 social workers responded (38.9% response rate). In a previous publication, the implications of automation for employee motivation were examined using the full sample.^26^ For the purpose of the present study, we use a subsample of social workers employed in financial aid (*n* = 1428), which is the area in social work where automation is most common. The subsample was used to ensure that the social workers had similar work tasks and used similar type of automation. The participants were divided into two groups: one group consisting of employees working with automation in terms of automated decision-making support (*n* = 345) and one group working without automated decision-making support (*n* = 1083). Automated decision-making support in the Swedish social services refers to algorithmic systems that assist human caseworkers in assessing eligibility, processing information, and making administrative decisions about welfare benefits. These systems typically analyse structured data (e.g., income, household composition, prior benefits) and generate recommendations, predictions, or preliminary assessments that inform—but do not replace—the final decision made by a human official. In the context of Swedish social services, automated decision-making support often takes the form of tools such as robotic process automation (RPA) or decision-support algorithms that standardise parts of the case-handling process, reduce administrative workload, and increase consistency. However, decision authority generally remains with the social worker, making these systems distinct from fully automated decision-making where no human is involved.^27^

The group with automated decision-making support had a mean age of 40.9 years (*SD* = 11.4), 448 (87.7%) were women. The group without automated decision-making support had a mean age of 42.2 years (*SD* = 11.0), 792 (85.8%) were women. The average duration of employment at the present position was 7.2 years (*SD* = 6.2) for those with automated decision making and 7.5 years (*SD* = 6.2) for those without.

The survey was distributed both via postal mail and through a digital mailbox. This was followed by friendly reminders a couple of weeks later. Distribution and administration of the questionnaire was carried out in cooperation with Statistics Sweden (SCB). SCB provided an attrition analysis based on their registers, concluding that higher age, female gender, having Sweden as country of birth, single or living together without children, and residents in sparsely populated areas were associated with a higher probability of answering our survey. The study was approved by the national ethical review authority (Ethical review number blinded for review).

## Measures

### Automation at work

Respondents were first provided with the following instructions: “*The following questions concern your experience with and attitude towards automation, which refers to systems that use robots or AI. These systems may include algorithms, also known as robots, that can collect and manage client information or utilize AI for more advanced tasks. Automation can be used, for example, as decision support in the processing of financial aid applications or in systems that automate administrative tasks, such as organizing cases*” and then answered the question “*Do you use automation in the form of robots or AI in systems at your workplace?*” with three response options: 1 = yes, 2 = no, but there are plans to implement; and 3 = no, and there are no plans to implement. The variable was dichotomized into 0 = no and 1 = yes.

**Work characteristics** were measured using validated scales selected based on their documented relevance for how automation influences the work environment**.** Scales were selected to represent all dimensions in the SMART model of work design.^6^ All scales used Likert-type response format.

Stimulating work characteristics were measured in terms of skill variety and use from Morgeson & Humphrey,^8^ assessed with four items. An example item is “My job requires a variety of skills”. Responses were rated on a 5-point scale ranging from 1 (strongly disagree) to 5 (strongly agree). The internal consistency (α) for this scale was .85.

The Mastery dimension is represented by Role clarity assessed with four items developed by Haynes et al.,^28^ (e.g., “I know what my responsibilities are”) using the same 5-point scale. The internal consistency (α) for this scale was .82.

The dimension including autonomous work characteristics was measured by decision-making autonomy assessed with three items from Morgeson & Humphrey.^8^ (e.g., “My job allows me to make a lot of decisions on my own”) using the same 5-point scale. The internal consistency (α) for this scale was .79.

Relational aspects were measured by beneficiary contact and social support. Beneficiary contact was assessed with three items by Grant^29^ (e.g., “My job allows frequent communication with the people who benefit from my work”). The scale used the same 5-point scale and the internal consistency (α) for this scale was .86. Social support was assessed with four items from Clausen et al.^30^ (e.g., “To what extent can you get advice and guidance from your colleagues when you need it?”). Responses were rated on a 5-point scale ranging from 1 (very little) to 5 (a great deal). The internal consistency (α) for this scale was .80.

The dimension tolerable work demands was represented by work demands and role conflict. Work demands were assessed with four items from Berthelsen et al.^31^ (e.g., “How often is your workload unevenly distributed so that work piles up?”) using a 5-point scale ranging from 1 (never) to 5 (always/almost always). The internal consistency (α) for this scale was .90. Role conflict was assessed with four items from Berthelsen et al.^32^ (e.g., “To what extent do you do things at work that are accepted by some people but not by others?”) using a 5-point scale ranging from 1 (To a very low extent) to 5 (To a very high extent). The internal consistency (α) for this scale was .70.

### Well-being

Vigour was assessed with three items developed by Schaufeli et al.^33^ (e.g., “At my work, I feel bursting with energy”). Responses were rated on a scale from 0 (never) to 6 (daily). The internal consistency (α) for this scale was .85.

Burnout was assessed with three items developed by Maslach et al.^34^ (e.g., “I feel emotionally drained by my work”). Responses were rated on a scale from 0 (never) to 5 (always). The internal consistency (α) for this scale was.82.

### Analysis

Analyses were performed with Mplus software, Version 8.9,^35^ and all models were estimated with full-information maximum likelihood. First, we conducted confirmatory factor analyses to estimate reliability and to build the measurement model in full as well as for employees without and with automated decision making separately.

Second, in line with recommendations, we examined measurement invariance of work characteristics and well-being to enable group comparisons.^36^ Established measures of model fit were applied: the comparative fit index (CFI), the root mean square of approximation (RMSEA) and the standardized root mean square residual (SRMR). Model fit was assessed using several model fit indices, including the chi-square fit index (χ2), RMSEA, SRMR, and CFI. In line with recommendations, acceptable fit was assumed when SRMR and RMSEA < .08 and CFI > . 90. To specify an excellent fit, cut-off criteria were more restrictive (SRMR and RMSEA < .08, CFI > .95^37^). Invariance was supported over groups when ΔCFI < .01, ΔRMSEA < .015 and ΔSRMR < .03 between the configural and metric model and between the metric and scalar model when ΔSRMR < .01.^38^

Finally, multigroup analyses were conducted to compare the latent means between the two groups. Latent mean differences between employees working without and with automated decision-making support were investigated by assessing the critical ratio (CR) index and effect size using Cohens D. A CR value equal to or greater than 1.96 was considered a significant difference in latent means^39^ and effect sizes were assessed as small (.10−.20), medium (.20−.40) or large (<.40) in line with Bosco et al.'s^40^ suggestions for research in applied psychology.

## Results

The latent means, standard deviations, and correlations between study variables are presented in Table 1.

**Table 1.** Descriptive statistics: latent means, standard deviations, and latent variable correlations between study variables for the two groups.

Variable	Latent mean no ADM	SD	1.	2.	3.	4.	5.	6.	7.	8.	9.	Latent mean ADM	SD
1. Skill variety and use	4.46	.50		.48	.41	.31	.20	.06 ns	.10 ns	.08ns	.07ns	4.45	.47
2. Role clarity	4.23	.53	.49		.59	.62	.40	−.28	−.32	.28	−.22	4.21	.53
3. Decision-making autonomy	4.22	.59	.50	.50		.48	.36	−.22	−.24	.13	−.18	4.19	.57
4. Beneficiary contact	4.01	.68	.44	.54	.41		.34	−.45	−.29	.39	−.36	3.89	.73
5. Social support	4.09	.60	.23	.39	.29	.26		−.22	−.20	.18	−22	4.01	.56
6. Work demands	2.95	.83	.05ns	−.20	−.11	−.34	−.29		.56	−.41	.66	3.06	.86
7. Role conflict	2.91	.57	.08	−.33	−.14	−.26	−.25	.50		−.36	.61	3.02	.59
8. Vigour	4.25	.71	.12	.36	.19	.29	.32	−37	−.37		−.58	4.22	.75
9. Burnout	2.78	.96	.06ns	−.21	−.09	−27	−.31	.68	.45	−.61		2.83	.93

*Note:* Employees working without ADM = 1083. Employees working with ADM = 345. SD = standard deviation. Correlations were significant at level p < .005 unless labelled ns (non-significant). Values presented to the left represent employees working without ADM and values to the right represent employees working with ADM.

Before comparing the two groups, we examined our measurement models, one for work characteristics and one for well-being, and both showed acceptable fit: χ^2^(278) = 1099.42, *p* < 0.05, RMSEA = 0.05, SRMR = 0.04, CFI = 0.95 for work characteristics and χ^2^(8) = 78.38, *p* < 0.05, RMSEA = 0.08, SRMR = 0.03, CFI = 0.98 for well-being. Next, we tested invariance between the group of employees working without and with automated decision-making support to ensure that our measures were perceived in a similar way in both groups. As presented in Table 2, invariance at the scalar level was supported, enabling further comparison of the two groups.

**Table 2.** Test of invariance.

Models	Chi square	df	RMSEA	SRMR	CFI	Model comparison	ΔRMSEA	ΔSRMR	ΔCFI
Work characteristics:									
Configural	1433	556	.047	.044	.949				
Metric	1465	575	.047	.047	.948	Configural vs. metric	.000	.003	.001
Scalar	1504	594	.046	.047	.947	Metric vs. scalar	.001	.001	.001
Well-being:									
Configural	98	16	.084	.039	.981				
Metric	104	20	.077	.039	.981	Configural vs. metric	.007	.000	.000
Scalar	108	24	.070	.040	.981	Metric vs. scalar	.007	.001	.000

*Note**:*** N = 1428, Employees working without ADM (*n* = 1083), employees working with ADM (*n* = 345).

We then compared the latent means of perceived work characteristics and well-being between employees without and with automated decision-making support, see Table 3. We found that employees working with automated decision-making support (ADM) reported statistically significantly lower levels of beneficiary contact (*M*_ADM_ = 3.89, *M*_no ADM_ = 4.01, Δ = -0.12, CR = −2.70), suggesting that employees working with automated decision-making support spend less time to meet and interact with clients compared to employees with no automated decision making. In addition, employees working with automated decision-making support also experienced lower levels of social support (*M*_ADM_ = 4.01, *M*_no ADM_ = 4.09, Δ = 0.09, CR = −2.13), and they reported higher levels of work demands (*M*_ADM_ = 3.06, *M*_no ADM_ = 2.95, Δ = 0.09, CR = 2.00). Further, employees working with automated decision-making support perceived higher levels of role conflict (*M*_ADM_ = 3.02, *M*_no ADM_ = 2.91, Δ = 0.09, CR = 2.47). The effect sizes were in general small, ranging from .13-.18, see Cohens D in Table 3. Regarding the work characteristics decision making autonomy, role clarity, skill variety and use no statistically significant difference was found between the groups. For the two well-being indicators, neither burnout nor vigour differed significantly between the two groups.

**Table 3.** Multigroup analysis. Comparison of latent means.

Construct	No ADM (mean)	ADM (mean)	Delta (Δ)	CR	Cohen's *d*
Skill variety and use	4.46	4.45	−0.004	−0.19	0.01
Role clarity	4.23	4.21	−0.017	−0.51	0.04
Decision-making autonomy	4.22	4.19	−0.023	−0.86	0.06
Beneficiary contact	4.01	3.89	−0.117	**−2**.**70**	**0**.**18**
Social support	4.09	4.01	−0.087	**−2**.**13**	**0**.**15**
Work demands	2.95	3.06	0.093	** 2**.**00**	**0**.**13**
Role conflict	2.91	3.02	0.087	** 2**.**47**	**0**.**18**
Vigour	4.25	4.22	−0.030	1.05	0.03
Burnout	2.78	2.83	0.053	−0.48	0.07

*Note:* CR = critical ratio. CR values in bold > 1.96 equals p < .05. Employees working without ADM (n = 1083), and with a ADM (n = 345).

## Discussion

The purpose of the present study was to examine how automation was related to work characteristics and well-being among social workers. Our analyses demonstrated that automation in the social services was associated with small detrimental differences on two aspects of work design, namely relational aspects and the perception of tolerable demands. No statistically significant differences were observed on social workers experience of how stimulating the job was or on perceptions of autonomy and mastery. In addition, we found no statistically significant differences in well-being among employees working with or without automation.

This study contributes to the growing field of automation at work by answering the call to examine how employees work characteristic may change due to the implementation of robots and AI at work.^3^ Based on theories of work design, automation may in theory have both positive or negative consequences for employees, depending on how it is implemented, and Demerouti^25^ argues that it is important to ensure that automation is turned into a resource to employees, not a demand, to ensure employee well-being. Our findings suggest that at least in the Swedish social service context, automation is first and foremost associated with negative consequences for perceived work characteristics, however, the observed differences were generally quite small.

Interestingly, we did not observe differences in all types of work characteristics, rather it was work characteristics related to relational aspects (such as social support and beneficiary contact) and perceived demands (such as quantitative demands and role conflict). This suggest that social workers who worked with automated decision-making support experienced less interaction with clients and colleagues, while at the same time had a higher workload and conflicting work tasks. This is in line with previous qualitative studies in the social work context, where social workers have raised concerns that automated decision making may reduce time with clients, while at the same time increasing the number of clients a social worker is expected to handle.^10, 15^ It is also in line with recent discussions in the field, that in particular relational aspects of work design are at risk when automation is introduced and collaboration may be with automated systems rather than with colleagues.^41^Our findings on increased demands also points towards the importance for organizations to actively turn automation into a resource, otherwise it may turn into a demand.^25^

No statistically significant differences were found on work characteristics related to stimulating, mastery or agency aspects of work design in relation to automation. This suggest that although the workload is higher and interactions with clients and colleagues are fewer, social workers in automated contexts still experience similar levels of skill variety, role clarity, and autonomy as colleagues in non-automated contexts. These findings may also explain why we did not observe any statistically significant differences in neither hedonic nor eudaimonic well-being between the two groups. The perception of sustained resources in some areas may buffer against the reduction of other resources such as support and increased perception of work demands and role conflict.^41^ In sum, our findings bring the field of automation at work forward by showcasing how work design theories may be helpful in highlighting the importance of work characteristics in understanding how professions may change due to automation.

### Practical implications

For organizations, the shift towards automation necessitates a re-evaluation of how work is organized and structured to secure employee work characteristics and well-being. Automation in social work presents both opportunities and challenges. By thoughtfully considering its impact on key work characteristics, organizations can implement automation in ways that enhance social workers’ roles and well-being. Our study particularly points towards the importance of preserving relational aspects at work while ensuring that work demands are tolerable in an automated context. This means, for example, to make sure that social workers, also after the implementation of automated tools, have enough time to meet with clients and to make sure that automation does not lead to more clients to handle.

In addition, our findings suggest that there are unlocked opportunities to turn automation into a resource for employees^25^ by using the potential of automation to increase social workers resources such as skill variety and autonomy as well as providing a clearer role. Automation may by taking over monotonous tasks,^9^ allow social workers to engage in diverse and complex activities that utilize both a broader skill set and enable them to focus on decision-making aspects of their role.^3^ Proactive work design, guided by established theories and empirical evidence, is essential to navigate this transformation effectively.^3^

### Limitations and future research

Although the present study has several strengths, such as the large national sample of social workers, it is not without limitations. This study's reliance on a cross-sectional design and self-reported data introduces potential limitations, particularly concerning common method bias (CMB). Cross-sectional designs capture data at a single point in time, which restricts the ability to infer causal relationships between variables.^42^Moreover, the use of self-reported measures raises concerns about CMB, where variance is attributable more to the measurement method than to the constructs being assessed.^43^ This means that we cannot be sure that our observed differences between groups are attributed to automation as it may reflect other underlying differences between our two groups. We implemented several procedural remedies, such as assuring anonymity and counterbalancing question order, to alleviate the potential biases inherent in self-reported data.^43^ In addition, as we compare latent means rather than assessing relationship between variables, the invariance across groups suggest that our latent mean comparison was robust. However, our findings should be viewed in light of these limitations and to further mitigate these issues, future research should consider longitudinal designs to better establish temporal precedence and causality as well as incorporating multiple data sources to minimize the risk of CMB. Also, while we chose to focus on specific work characteristics that had been suggested in previous research, there may be other work characteristics that are affected by automation as well. Future studies may therefor examine additional work characteristics in relation to automation.

Future research is also needed to replicate our findings among social workers in other countries and other highly educated professions to examine the robustness of our findings. For example, future studies may test if relational aspects and the perception of work demands are particularly affected by automation. The replication of our findings is particularly important given the fast development in the area of automation, as updated products or services in combination with the increased experience employees have using these systems may change the impact automation have on work characteristics. Additionally, future studies could explore potential moderators that may increase or reduce the impact of automation on work characteristics and employee wellbeing, such as type of automation, employees’ technological readiness as well as individual differences and employee motivation.^44^ By uncovering potential moderators, deeper insights will be provided into how different groups of employees’ experience automation, which may to helpful to identify targeted interventions to support those most affected by automation-related challenges.^3^

## Conclusion

In conclusion, our study contributes to the growing field of automation at work^1, 9^ by drawing on theories of work design^3^ to disentangle the potential positive and negative consequences of automation for employees work characteristics and well-being. Our findings suggest that automation among social workers in particular may have negative consequences for relational aspects and the perception of work demands, while not necessarily affecting employee well-being. Our findings are a first step towards a fuller understanding of how automation is perceived by highly educated employees in the public sector and suggest that organizations that strive to implement automated work process should be aware of the potential consequences such work procedures have on employees.

## Data Availability

Data are available from the first author upon reasonable request.
